# Antiretroviral drug use and HIV drug resistance in female sex workers in Tanzania and the Dominican Republic

**DOI:** 10.1371/journal.pone.0240890

**Published:** 2020-10-29

**Authors:** Wendy Grant-McAuley, Jessica M. Fogel, Noya Galai, William Clarke, Autumn Breaud, Mark A. Marzinke, Jessie Mbwambo, Samuel Likindikoki, Said Aboud, Yeycy Donastorg, Martha Perez, Clare Barrington, Wendy Davis, Deanna Kerrigan, Susan H. Eshleman

**Affiliations:** 1 Department of Pathology, Johns Hopkins University School of Medicine, Baltimore, Maryland, United States of America; 2 Department of Epidemiology, Johns Hopkins University School of Public Health, Baltimore, Maryland, United States of America; 3 Department of Statistics, University of Haifa, Mt Carmel, Israel; 4 Department of Psychiatry, Muhimibili University of Health and Allied Sciences, Dar es Salaam, Tanzania; 5 Department of Microbiology and Immunology, Muhimibili University of Health and Allied Sciences, Dar es Salaam, Tanzania; 6 Unidad de Investigacion de Vacunas, Instituto Dermatologico y Cirugia de la Piel, Santo Domingo, Dominican Republic; 7 Department of Health Behavior, University of North Carolina, Chapel Hill, North Carolina, United States of America; 8 Center on Health, Risk and Society, American University, Washington, District of Columbia, United States of America; University of Cincinnati College of Medicine, UNITED STATES

## Abstract

**Objective:**

Female sex workers (FSW) have increased risk of HIV infection. Antiretroviral treatment (ART) can improve HIV outcomes and prevent HIV transmission. We analyzed antiretroviral (ARV) drug use and HIV drug resistance among HIV-positive FSW in the Dominican Republic and Tanzania.

**Methods:**

Plasma samples collected at study entry with viral loads >1,000 copies/mL were tested for ARV drugs and HIV drug resistance. ARV drug testing was performed using a qualitative assay that detects 22 ARV drugs in five classes. HIV genotyping was performed using the ViroSeq HIV-1 Genotyping System. Phylogenetic analyses were performed to determine HIV subtype and assess transmission clusters.

**Results:**

Among 410 FSW, 144 (35.1%) had viral loads >1,000 copies/mL (DR: n = 50; Tanzania: n = 94). ARV drugs were detected in 36 (25.0%) of 144 samples. HIV genotyping results were obtained for 138 (95.8%) cases. No transmission clusters were observed in either country. HIV drug resistance was detected in 54 (39.1%) of 138 samples (31/35 [88.6%] with drugs detected; 23/103 [22.3%] without drugs detected); 29/138 (21.0%) had multi-class resistance (MCR). None with MCR had integrase strand transfer inhibitor resistance. In eight cases, one or more ARV drug was detected without corresponding resistance mutations; those women were at risk of acquiring additional drug resistance. Using multivariate logistic regression, resistance was associated with ARV drug detection (p<0.001), self-reported ART (full adherence [p = 0.034]; partial adherence [p<0.001]), and duration of HIV infection (p = 0.013).

**Conclusions:**

In this cohort, many women were on ART, but were not virally suppressed. High levels of HIV drug resistance, including MCR, were observed. Resistance was associated with detection of ARV drugs, self-report of ART with full or partial adherence, and duration of HIV infection. These findings highlight the need for better HIV care among FSW to improve their health, reduce HIV drug resistance, and decrease risk of transmission to others.

## Introduction

The HIV epidemic is a global public health crisis, with approximately 37.9 million people living with HIV and 1.7 million new infections per year [1]. Female sex workers (FSW) are at higher risk of HIV infection compared to women in the general population, with an overall global prevalence of 10.4% [2–4]. This disparity has been documented in sub-Saharan Africa and the Caribbean, where HIV prevalence among FSW is 36.9% and 6.1% respectively, compared to 7.4% and 0.38% among the general population of cisgender women [2, 3]. Female sex work also plays an important role in HIV transmission dynamics and is the probable source of ~15% of HIV infections in women worldwide [5]. In addition, data from more than 70 countries indicate that HIV prevalence among FSW serves as the greatest indicator of national HIV prevalence [6]. Research with FSW living with HIV has been limited by difficulties in study recruitment due to sex-work related stigma, population mobility, and criminalization of sex work [7–10]. Available data suggest that FSW across the globe struggle to engage effectively with programs for HIV diagnosis and treatment [11–15]. Poor engagement of FSW in care has been associated with several factors, including low socioeconomic status, gender discrimination, and stigma associated with HIV infection and sex work [13–16]. Ineffective HIV care and treatment negatively impact viral suppression in the FSW population, increasing the risk of HIV transmission to others [4, 13, 14].

HIV drug resistance can limit the ability to achieve viral suppression among HIV-positive people on antiretroviral therapy (ART) [17, 18]. Drug-resistant HIV can also emerge during ART, especially with suboptimal adherence, leading to treatment failure and limiting treatment options [17, 18]. Drug-resistant HIV can also be transmitted to others [18, 19]. To date, relatively few studies have evaluated ART failure and HIV drug resistance among FSW [11, 12, 19, 20]. Many FSW are not virally suppressed, despite high levels of self-reported ART adherence [12, 19, 20], and a high prevalence of drug resistance has been observed in HIV-positive FSW regardless of their treatment status [19, 20]. Data on ARV drug use among FSW are also limited and have been based primarily on self-report [19, 20], which may be unreliable [21–25]. Participants in research studies may over-report ARV drug use for a variety of reasons (e.g., because they misunderstand questionnaires or prefer to give socially-desirable answers) [25, 26]. ARV drug use may also be underreported in order to meet study eligibility requirements or to hide prior knowledge of HIV status [27]. Some individuals may also use ARV drugs for reasons other than ART, such as pre-exposure prophylaxis (PrEP, in those who are not aware that they are infected), hepatitis treatment, or recreational use [28, 29]; these data would not be captured in questionnaires that ask only about ARV drug use for HIV treatment.

Given the heightened burden of disease among FSW and their integral role in the on-going global HIV epidemic, there is a clear need for a comprehensive evaluation of ARV drug use and HIV drug resistance among FSW living with HIV. In this study, we evaluated ARV drug use and HIV drug resistance among FSW in two geographically and epidemically distinct settings.

## Methods

### Study cohort

FSW living with HIV were recruited for study participation in two areas with high HIV burden among FSW: the Iringa region of Tanzania and the Dominican Republic (DR). Participants were enrolled in 2017 and 2018 in a study focused on social determinants of HIV outcomes. Participants in the DR (Abriendo Puertas [13]) were recruited largely through use of peer navigators, while those in Tanzania (Project Shikamana [14]) were recruited using venue time-location sampling. Plasma samples and social demographic, behavioral, and clinical data were collected from participants at enrollment and at six- and twelve-month follow-up visits. In this study, ARV drug testing and HIV genotyping were performed using baseline samples from participants who had viral loads >1,000 copies/mL at study entry.

### Laboratory testing

Viral load testing was performed at the at the study sites (Muhimbili University of Health and Allied Sciences [MUHAS] in Iringa, Tanzania; Instituto Dermatológico y Cirugía de la Piel [IDCP] in Santo Domingo, DR) using the Roche Amplicor HIV-1 Monitor test [13, 14]. All other testing was performed retrospectively at Johns Hopkins University (Baltimore, MD). Plasma samples were analyzed using a qualitative multi-drug assay based on high-performance liquid chromatography coupled with high resolution mass spectrometry [30]. This assay detects 22 ARV drugs in five drug classes (nine protease inhibitors [PIs], six nucleoside/nucleotide reverse transcriptase inhibitors [NRTIs], three non-nucleoside reverse transcriptase inhibitors [NNRTIs], three integrase strand transfer inhibitors [INSTIs], and one CCR5 receptor antagonist); analyte lower limits of detection are drug-specific and range from 2–20 ng/mL. HIV genotyping was performed using the ViroSeq HIV-1 Genotyping System, v2.0 (Abbott Molecular, Des Plaines, IL); this assay produces a 1302 base pair sequence which encodes HIV protease and the first 335 amino acids of HIV reverse transcriptase. Samples with both NRTI and NNRTI resistance were also tested using the ViroSeq HIV-1 Integrase Genotyping Kit, RUO (Abbott Molecular, Des Plaines, IL); this assay produces an 864 base pair sequence encoding HIV integrase. HIV drug resistance was assessed using the ViroSeq HIV-1 Genotyping Software, v.3.0 and ViroSeq Integrase Software 1.0.

### Phylogenetic analysis

HIV subtyping was performed using two automated online tools (REGA HIV Subtyping tool v3.0 and the Recombination Identification Program); HIV subtypes were confirmed by phylogenetic analysis using FastTree v2.1.10 with HIV subtype reference sequences from the Los Alamos National Laboratory’s HIV Sequence Database [31]. For cases with conflicting subtype results, subtype was assigned based on agreement using two of three subtyping methods.

Phylogenetic trees were constructed for cluster analysis using HIV *pol* sequences from study samples. Up to ten similar background sequences per study sequence were identified with BLAST [32]. After duplicate sequences were removed, the remaining background sequences were obtained from the LANL HIV Sequence Database [31]. Recombination breakpoints were identified with RDP4 [33]; sequences with breakpoints were excluded from the analysis. Multiple pairwise sequence alignment was performed with MAFFT v6.864 [34]. Trees were constructed with the randomized accelerated maximum-likelihood (RAxML) v8.2.12 method, accessed via the CIPRES Science Gateway [35]. Potential clusters were identified with Cluster Picker [36], using a maximum genetic distance threshold setting of 4.5% and a bootstrap support value threshold setting of ≥90% [37]. Phylogenetic tree graphics were created with the Interactive Tree of Life (iTOL) software v5.6.3 [38].

### Statistical analysis

In univariate analyses, social demographic, behavioral, and clinical factors associated with HIV drug resistance were evaluated using Chi-square tests and simple logistic regression, stratified by country and combined. The initial multivariate model included covariates that were either p<0.3 in the univariate analyses or hypothesized to be potentially related to resistance. The final multivariate logistic regression model was determined by applying backward stepwise procedures. The same methods were applied for stratification by country.

### Ethical considerations

Verbal informed consent was obtained from all individuals who were screened for participation in the parent study; all participants were compensated ~$USD5 at each study visit. The study was approved by the institutional review boards of MUHAS Health and the National Institute for Medical Research in Tanzania, the IDCP in the DR, and the Johns Hopkins Bloomberg School of Public Health in the United States.

### GenBank accession numbers

GenBank accession numbers for HIV sequences reported in this study are MN978752—MN978889 (HIV protease/RT) and MN978890—MN978916 (HIV integrase).

## Results

### Samples analyzed

**Fig 1:**
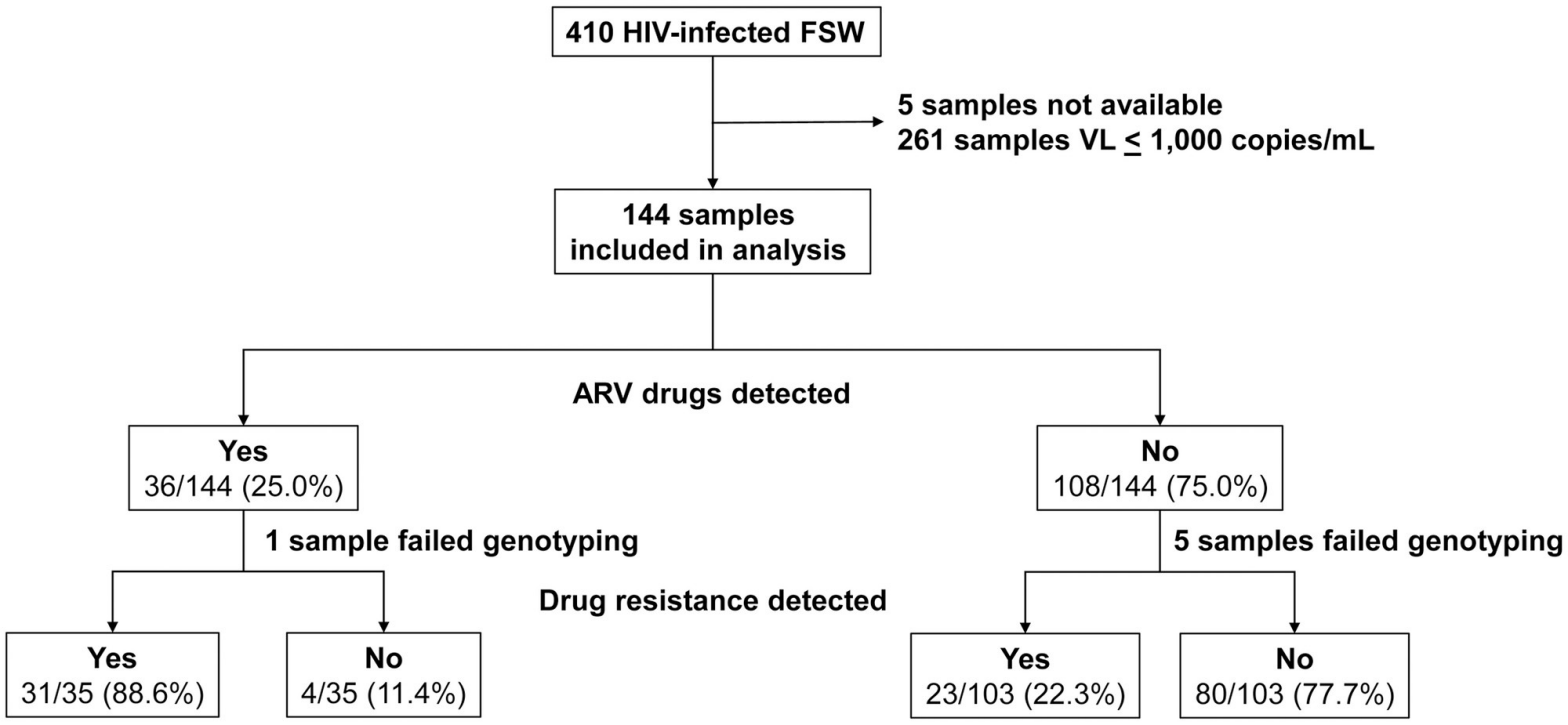
Overview of antiretroviral drug testing and HIV drug resistance testing. The figure shows an overview of the samples used for testing and a summary of the test results. Abbreviations: FSW: female sex workers; VL, viral load; mL, milliliter; ARV, antiretroviral.

The parent study enrolled 410 HIV-positive FSW (209 from Tanzania, 201 from the DR). Plasma samples collected at enrollment were available for 405 (98.8%) of the 410 participants (204 from Tanzania, 201 from the DR). HIV viral load was >1,000 copies/mL at enrollment for 144 (35.6%) of the 405 participants (94/204 [46.1%] from Tanzania, 50/201 [24.9%] from the DR); these samples were included in the analysis of ARV drug use and HIV drug resistance (Fig 1).

### HIV phylogenetic analysis

**Fig 2:**
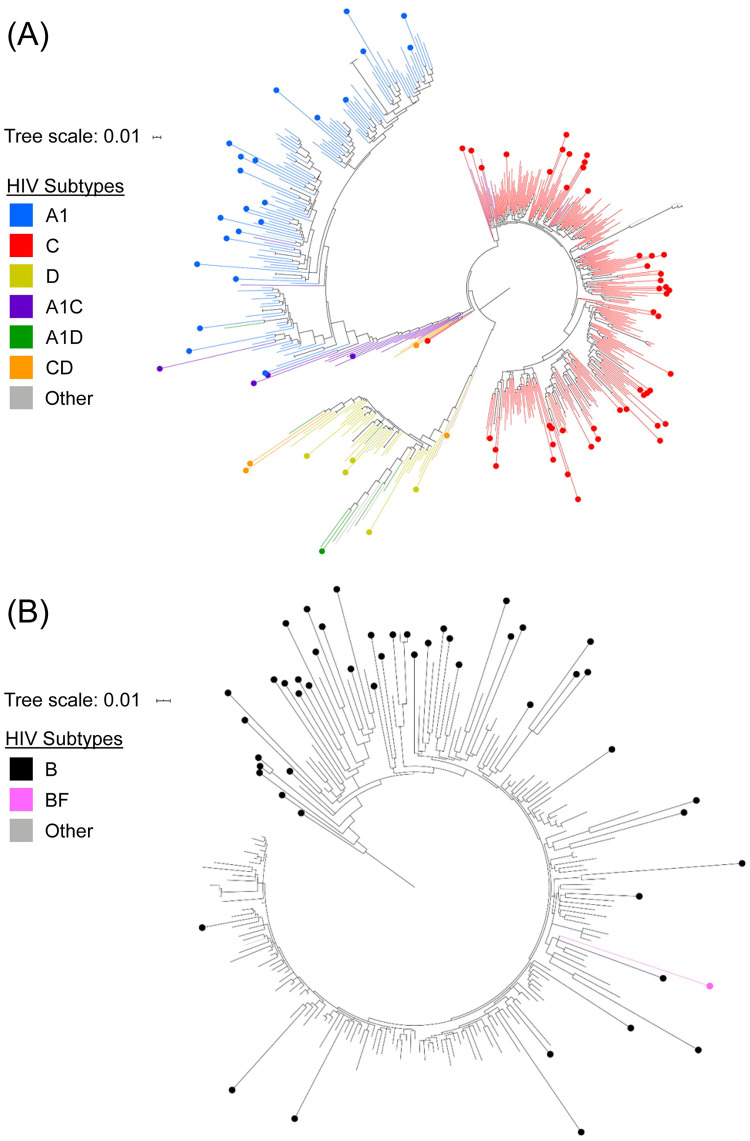
Phylogenetic trees of HIV *pol* sequences from Tanzania (Panel A), and the Dominican Republic (Panel B). Phylogenetic trees were constructed using study sequences from Tanzania (Panel A) and the Dominican Republic (Panel B) to identify potential transmission clusters. Dots at branch tips denote study sequences; plain branch tips denote background sequences. Sequences are color-coded according to HIV subtype. Background sequences that were most similar to study sequences from Tanzania were from Uganda (17.7%), Tanzania (14.2%), and South Africa (11.5%). Background sequences that were most similar to study sequences from the Dominican Republic were from the United States (66.7%), Spain (10.3%), and Canada (3.6%); only one background sequence originated from the Dominican Republic. There was no evidence of transmission clusters among sequences from either site, using a maximum genetic distance threshold of 4.5% and a bootstrap support value threshold of ≥90%.

HIV subtypes were determined by phylogenetic analysis of *pol* region sequences obtained from HIV genotyping. HIV genotyping results were obtained for 138 (95.8%) of the 144 cases (88 from Tanzania, 50 from the DR). The subtypes detected in Tanzania were C (55.7%); A1 (25.0%); CD recombinant (6.8%); D (5.7%); and A1C recombinant (5.7%). One sample was an A1D recombinant (1.1%). In the DR, 98.0% were subtype B, and one sample (2.0%) was a BF recombinant. Transmission cluster analysis was performed separately for each country (Fig 2). The analysis included 85 study sequences and 480 background sequences from Tanzania and 50 study sequences and 165 background sequences from the DR. The study sequences were dispersed among the background sequences with no evidence of transmission clusters in either country.

### Detection of ARV drugs

**Fig 3:**
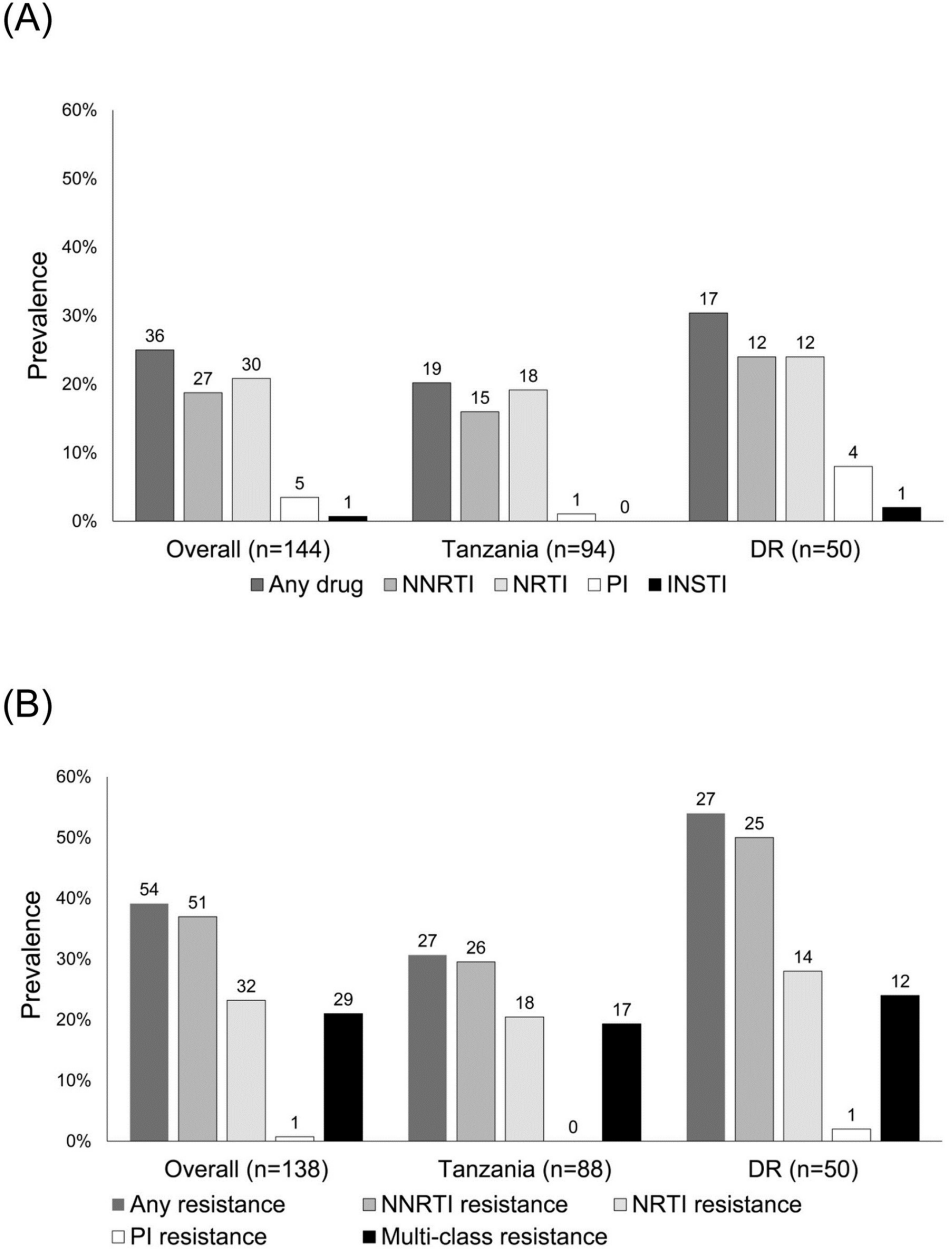
Detection of antiretroviral drugs and HIV drug resistance in samples from female sex workers with viral loads >1,000 copies/mL. The figure shows a summary of results from antiretroviral (ARV) drug testing (Panel A) and HIV drug resistance testing (Panel B). Results are shown for each site and for the two sites combined. Numbers above each bar indicate the number of samples with a positive test result (Panel A: one or more ARV drug detected; Panel B: one or more major drug resistance mutation detected). Abbreviations: DR: Dominican Republic; NNRTI: non-nucleoside reverse transcriptase inhibitor; NRTI: nucleoside/nucleotide reverse transcriptase inhibitor; PI; protease inhibitor; INSTI: integrase strand transfer inhibitor.

At least one ARV drug was detected in samples from 36 (25.0%) of 144 participants (19/94 [20.2%] from Tanzania, 17/50 [34.0%] from the DR, Figs 1 and 3). Table 1 shows the data from the 36 samples with ARV drugs detected. NNRTIs were detected in 27 (18.8%) of the samples, NRTIs were detected in 30 (20.8%) of the samples, and PIs were detected in five (3.5%) of the samples. An INSTI was detected in one sample from the DR. Of note, three samples from Tanzania had one or more NRTIs detected alone; one had lamivudine, one had lamivudine and zidovudine, and one had lamivudine, stavudine, and zidovudine.

**Table 1:** Antiretroviral drugs and major drug resistance mutations detected in samples from female sex workers with viral loads >1,000 copies/mL.

#	Site	ARV drugs detected			Major drug resistance mutations detected		
		NNRTI	NRTI	PI/INSTI	NNRTI	NRTI	PI
**1**	TZ		FTC	ATV	K103N	M184V	^a^
**2**	TZ	EFV	3TC, TFV		L100I, K101E, G190A	K65R, M184V, *K219E*	
**3**	TZ	EFV	3TC, TFV		L100I, K103N	K65R, M184V	
**4**	TZ	EFV	3TC, TFV		K103N	*D67N, K70R*, M184V, *K219E*	
**5**	TZ	EFV	3TC		L100I, Y181C, G190A	*D67N, K70R*, L74I, M184V, *T215F, K219E/Q*	
**6**	TZ	EFV	3TC		K103N, Y181C, G190A	*K70R*, M184V, *K219E/Q*	
**7**	TZ	EFV	3TC		L100I, K103N	K65R, M184V, *T215Y*	
**8**	TZ	EFV	3TC		K103N, Y181C	M184V, *T215F*	
**9**	TZ	EFV	3TC		K103N	K70E, M184V	
**10**	TZ	EFV	3TC		K103N	M184V	
**11**	TZ	EFV			K103N, Y181C	M184V	
**12**	TZ	NVP	3TC, ZDV		K101E, G190A	*M41L, K70R*, M184V, *T215Y*	
**13**	TZ	NVP	3TC		K103N, Y181C	M184V	
**14**	TZ		3TC, d4T, ZDV		K103N	M184V	
**15**	TZ		3TC, ZDV		K103N	M184V	
**16**	TZ		3TC			M184V	
**17**	DR		3TC	LPV, RTV	Y188L, G190A	*D67N, K70R*, M184V, *K219E*	V82A, L90M
**18**	DR	EFV	3TC		K103N	*K70R*, M184V	L90M
**19**	DR	EFV	3TC, TFV		K101E, Y181C, G190S	K65R, M184V	
**20**	DR	NVP	3TC, ZDV		Y188L	*M41L*, M184V, *L210W, T215Y*	
**21**	DR	EFV	ABC, 3TC		K103N, Y188L	*M41L, D67N, K70R*, M184V, *T215F, K219Q*	
**22**	DR	EFV			K101E, G190S	K65R, M184V	
**23**	DR	EFV			K103N	K65R, L74I, M184V	
**24**	DR	EFV	3TC, TFV		K103N, Y181C, G190A	L74I, M184V, *T215F*	
**25**	DR	EFV			K103N, G190A	M184V	
**26**	DR	NVP	3TC		Y181C	*M41L*, M184V, *L210W, T215Y*	
**27**	DR	EFV			K103N		
**28**	DR	EFV	3TC, TFV		K103N	^a^	
**29**	DR	EFV			K101E, K103N, G190A		
**30**	DR		3TC	LPV, RTV		M184V	^a^
**31**	DR		3TC, TFV	RLV		M184V	^a^
**32**	TZ	EFV	3TC, TFV		^a^	^a^	
**33**	TZ	EFV	3TC, TFV		^a^	^a^	
**34**	TZ	EFV	3TC			Failed genotyping	
**35**	DR		FTC, TFV	ATV, RTV		^a^	^a^
**36**	DR		3TC, TFV	ATV, RTV		^a^	^a^
**37**	TZ				K103N	M184V	
**38**	TZ				K103N		
**39**	TZ				K103N		
**40**	TZ				K103N		
**41**	TZ				K103N		
**42**	TZ				K103N		
**43**	TZ				K103N		
**44**	TZ				G190E	*D67N*	
**45**	TZ				K103N		
**46**	TZ				K103N		
**47**	TZ				K103N		
**48**	DR				K103N	K65R, M184V	
**49**	DR				L100I, K103N	K65R	
**50**	DR				K103N		
**51**	DR				V106M		
**52**	DR				K103N		
**53**	DR				K103N		
**54**	DR				K103S		
**55**	DR				K103R		
**56**	DR				L100I, K103N		
**57**	DR				K103N		
**58**	DR				Y181C		
**59**	DR				L100I, K103N		

The table shows the pattern of ARV drugs and HIV drug resistance mutations detected in the subset of 59 samples that were positive with one or both assays. Resistance mutations shown in italics are thymidine analog mutations (TAMs).

^a^ Indicates the risk for developing additional resistance (detection of one or more ARV drugs without the corresponding resistance mutations).

Abbreviations: 3TC, lamivudine; ABC, abacavir; ATV, atazanavir; ARV, antiretroviral; d4T, stavudine; DR, Dominican Republic; EFV, efavirenz; FTC, emtricitabine; LPV, lopinavir; NNRTI, non-nucleoside reverse transcriptase inhibitor; NRTI, nucleoside/nucleotide reverse transcriptase inhibitor; NVP, nevirapine; RLV, raltegravir; RTV, ritonavir; TFV, tenofovir; TZ, Tanzania; ZDV, zidovudine.

Among 78 of the participants who reported that they were on ART, only 35 (44.9%) had ARV drugs detected (18/41 [43.9%] from Tanzania, 17/37 [45.9%] from the DR). In addition, one (1.5%) of 66 FSW who reported that they were not on ART had ARV drugs detected (efavirenz and lamivudine; 1/53 [1.9%] from Tanzania, 0/13 [0%] from the DR). Of the 35 women who reported they were on ART, 29 were considered adherent to ART based on a cumulative score from four questions regarding adherence. Of those 29 women, only 18 (62.1%) had ARV drugs detected (7/13 [53.8%] from Tanzania and 11/16 [68.8%] from the DR).

### Analysis of HIV drug resistance

HIV drug resistance was detected in 54 (39.1%) of 138 cases (27/88 [30.7%] from Tanzania, 27/50 [54.0%] from the DR; Fig 2). NNRTI resistance was detected in 51 (37.0%) of 138 cases (26/88 [29.5%] from Tanzania, 25/50 [50.0%] from the DR). NRTI resistance was detected in 32 (23.2%) of 138 cases (18/88 [20.5%] from Tanzania, 14/50 [28.0%] from the DR). PI resistance was detected in one case from the DR (0.7%). Multi-class resistance (MCR; resistance to more than one ARV drug class) was detected in 29 (21.0%) of 138 cases (17/88 [19.3%] from Tanzania, 12/50 [24.0%] from the DR). Of these 29 cases, all had NNRTI and NRTI resistance; one case from the DR also had PI resistance. INSTI resistance was assessed for the 29 samples with MCR; none of these samples had INSTI resistance.

Table 1 shows the patterns of major drug resistance mutations detected in the 54 samples noted above. Forty-seven (87.0%) of the 54 samples had the NNRTI resistance mutations K103N, Y181C, Y188L, or G190A/S mutations alone or in combination. Thirty-one (57.4%) of the 54 samples had the NRTI resistance mutations M184V or K65R alone or in combination. Many samples also had one or more thymidine analog mutation (TAM) detected (n = 13). PI resistance mutations were detected in two (3.7%) of the 54 samples (one had V82A and L90M; one had L90M alone). No INSTI resistance mutations were detected.

### Relationship between ARV drug use and HIV drug resistance

The prevalence of HIV drug resistance was higher among the participants with ARV drugs detected compared to those with no drugs detected (31/35 [88.6%] vs. 23/103 [22.3%], p<0.001; Fig 1, Table 2). In most cases, the mutations detected corresponded to the drugs detected in the same sample. Of note, only two of those with a TAM had zidovudine detected, and none had stavudine detected. HIV from eight samples with one or more ARV drugs detected lacked corresponding resistance mutations, indicating that those women were at risk of acquiring additional drug resistance.

**Table 2:** Factors associated with HIV drug resistance among female sex workers with viral loads >1,000 c/mL.

Characteristics	Total n = 138	Univariate				Multivariate		
		Resistance Detected		OR	P-value	AOR	95% CI	P-value
		No n = 84	Yes n = 54					
**Study site**								
** Tanzania**	88	61 (69.3%)	27 (30.7%)	0.38	**0.007**			
** Dominican Republic**	50	23 (46.0%)	27 (54.0%)	1.00				
**Duration of HIV infection, mean (SD)**	5.2 (5.3)	3.7 (4.3)	7.8 (5.8)	1.17	**<0.001**	1.14	1.03, 1.27	**0.013**
**Currently on ART (self-report)**					**<0.001**			
** No**	62	58 (93.6%)	4 (6.5%)	1.00				
** Yes**	76	26 (34.2%)	50 (65.8%)	27.88				
**Self-reported adherence level**^a^					**<0.001**			
** No ART/No adherence**^b^	71	63 (88.7%)	8 (11.3%)	1.00				
** Partial adherence**	39	12 (30.8%)	27 (69.2%)	17.72		13.37	3.35, 53.37	**<0.001**
** Full adherence**	28	9 (32.1%)	19 (67.9%)	16.62		6.14	1.15, 32.80	**0.034**
**ARV drugs detected in blood**					**<0.001**	12.79	3.35, 48.77	**<0.001**
** No**	103	80 (77.7%)	23 (22.3%)	1.00				
** Yes**	35	4 (11.4%)	31 (88.6%)	26.96				
**Age in years, mean (SD)**	31.7 (8.4)	29.2 (6.4)	35.9 (9.9)	1.11	**<0.001**			
**Marital status, single**					**0.001**			
** No**	78	38 (48.7%)	40 (51.3%)	1.00				
** Yes**	60	46 (76.7%)	14 (23.3%)	0.29				
**Number of live births**					**0.005**			
** 0**	12	9 (75.0%)	3 (25.0%)	1.00				
** 1-2**	77	54 (70.1%)	23 (29.9%)	1.28				
** 3+**	49	21 (42.9%)	28 (57.1%)	4.00				
**Seen provider at ANC last pregnancy**					**0.005**			
** No**	23	20 (87.0%)	3 (13.0%)	1.00				
** Yes**	115	64 (55.7%)	51 (44.4%)	5.31				
**Travel (past 6 months)**					0.263			
** No**	84	48 (57.1%)	36 (42.9%)	1.00				
** Yes**	54	36 (66.7%)	18 (33.3%)	0.67				
**Number new/regular clients (past 30 days)**					0.295			
** ≤4**	69	45 (65.2%)	24 (34.8%)	1.00				
** >4**	69	39 (56.5%)	30 (43.5%)	1.44				
**Inconsistent condom use with new/regular clients (past 30 days)**					**0.028**			
** No**	84	45 (53.6%)	39 (46.4%)	1.00				
** Yes**	54	39 (72.2%)	15 (27.8%)	0.44				
**Alcohol (≥4 days per week)**					0.774			
** No**	90	54 (60.0%)	36 (40.0%)	1.00				
** Yes**	48	30 (62.5%)	18 (37.5%)	0.92				
**Drug use (ever)**					0.375			
** No**	110	69 (62.7%)	41 (37.3%)	1.00				
** Yes**	28	15 (53.6%)	13 (46.4%)	1.46				
**Sex work stigma**^c^								
** <36**	91	58 (63.7%)	33 (36.3%)	1.00	0.337			
** ≥36**	47	26 (55.3%)	21 (44.7%)	1.42				
**Gender-based violence (past 6 months)**					0.823			
** No**	91	56 (61.5%)	35 (38.5%)	1.00				
** Yes**	47	28 (59.6%)	19 (40.4%)	1.09				

The table shows factors associated with HIV drug resistance in univariate models and a multivariate model generated by backwards selection (see text); the multivariate model included data from 132 cases. The initial model used for multivariate analysis included the following variables: country, age, being single, travel in the last 6 months, having ≥3 live births, visiting an ANC during last pregnancy, having >4 clients per week on average, consistent condom use, HIV duration, self-reported ART adherence level, and detection of ARV drugs. Self-reported current ART correlated with self-reported ART adherence and therefore was not included in the initial multivariate model.

^a^ The level of self-reported ART adherence was the sum of four adherence measures (adherence in last 4 days, always take on schedule, always follow instructions, and not skipped last weekend). Responses were scored as full adherence (4), partial adherence (1–3), no ART/no adherence (0).

^b^ This included nine participants who reported that they were “currently on ART”.

^c^ The stigma score was calculated as the sum of a 13-item Likert type scale [14].

Abbreviations: OR, odds ratio; CI, confidence interval; SD, standard deviation; ANC, antenatal clinic; STI, sexually transmitted infection; ART, antiretroviral treatment; ARV, antiretroviral.

### Factors associated with HIV drug resistance

We examined the relationship between HIV drug resistance and social demographic, behavioral, and clinical factors (Table 2). Univariate analysis demonstrated that resistance was associated with study site (higher prevalence in the DR), older age, marital status, higher number of live births, engagement at an antenatal clinic (ANC) during the last pregnancy, consistent condom use, longer duration of HIV infection, current ART by self-report, self-reported partial or full ART adherence, and detection of ARV drugs. There was no significant association between resistance and recent travel, number of new clients, alcohol or substance use, sex-work stigma, or gender-based violence.

In a multivariate logistic model, HIV drug resistance was independently associated with longer duration of HIV infection (adjusted odds ratio [aOR]: 1.14, 95% confidence interval [CI]: 1.03, 1.27), self-reported partial (aOR: 13.37, 95%CI: 3.35, 53.37) or full ART adherence (aOR: 6.14, 95%CI: 1.15, 32.80) (vs. no ART/no adherence), and detection of ARV drugs (aOR: 12.79, 95%CI: 3.35, 48.77). The model had an area under the curve (AUC, or c-statistics) of 91.2%, indicating a very high ability to discriminate between cases with vs. without resistance. In multivariate models stratified by site, duration of HIV infection and detection of ARV drugs were independently associated with resistance at both sites. Self-reported partial ART adherence was independently associated with resistance in Tanzania only (S1 Table).

## Discussion

This study evaluated patterns of ARV drug use and HIV drug resistance in FSW in Tanzania and the DR. At enrollment, over one third of the women had viral loads >1,000 copies/mL and one quarter of those women had ARV drugs detected in study samples. The drug combinations detected mostly reflected first-line ART regimens used in each country at the time the study was performed (2 NRTIs + 1 NNRTI or 2 NRTIs + 1 PI) [39–41]. In Tanzania, first-line drugs included zidovudine, stavudine, lamivudine, emtricitabine, tenofovir, nevirapine, and efavirenz [39]; in the DR, first-line drugs included zidovudine, lamivudine, nevirapine, and efavirenz [41]. At the time of this study, universal ART was not available in the DR; instead, ART initiation was based on CD4 cell count and other AIDS-defining criteria [41]. The DR has since adopted universal ART [42] with dolutegravir-based first-line regimens [43].

Only one participant had an INSTI detected. In Tanzania, three participants had one or more NRTIs detected without drugs from other classes. This may reflect sub-optimal ART adherence or continued use of regimens previously used in sub-Saharan Africa, including triple NRTI regimens [44, 45]. Notably, only 45% of those who reported that they were currently on ART had ARV drugs detected. Similar discrepancies between self-reported ART and objective measures of ARV drug use using laboratory testing have been reported in other cohorts and settings [25, 46, 47]. For example, in a retrospective analysis of a subset of participants from the HPTN 052 study, 29% of participants who self-reported being ARV drug naive had ARV drugs detected [25]. In the HPTN 074 study, ARV drugs were detected in only 75% of those who self-reported being on ART and in 8% of those who reported not being on ART [46].

HIV drug resistance was detected in ~40% of those tested and many had resistance to more than one class of ARV drugs. INSTI resistance was not observed among any of those with MCR. Drug resistance was most strongly associated with detection of ARV drugs and self-reported full or partial adherence to ART (vs. no ART/no adherence). Drug resistance was detected in nearly 90% of the FSW who had ARV drugs detected. This is higher than the proportion that has been observed in other cohorts and settings [22, 23, 48]. Further studies are underway using quantitative ARV assays to evaluate whether HIV drug resistance among FSW is associated with sub-optimal ART adherence.

The frequency of virologic failure, HIV drug resistance, and MCR were all higher in this cohort compared to data from a recent, smaller study of FSW from Uganda [19]. In this study, eight participants were also identified who were at risk of developing additional drug resistance. These findings indicate that treatment options for many FSW are limited. The low prevalence of PI resistance and the lack of detection of INSTI resistance in this cohort suggest that PI- and INSTI-based regimens, including those with long-acting drugs, should be considered for HIV treatment in this population.

Drug resistance was also observed in samples from 22% of women in this study who did not have ARV drugs detected. This may reflect lapses in ART due to inconsistent access to care, but could also reflect prior exposure to ARV drugs for HIV treatment or other indications, or infection with drug-resistant HIV. Transmitted drug resistance was not assessed in this study since many FSW in this cohort were previously diagnosed with HIV (mean duration of infection: 5 years) and may have had prior exposure to ARV drugs. A recent study of a smaller cohort of FSW in Brazil found that approximately half of the participants who reported that they were ART-naïve had drug resistance mutations [49]. Further studies are needed to assess the frequency of transmitted HIV drug resistance among FSW.

Phylogenetic cluster analysis was also performed for each study site; this analysis revealed no evidence of HIV transmission networks. Sequences most similar to those from study participants in Tanzania were from several countries in sub-Saharan Africa. Given Iringa’s proximity to the Tanzam Highway and the known migratory aspect of sex work, it is likely that FSW from this region are involved in both local and international transmission networks [14]. Sequences are available from several studies focused on HIV transmission networks in Hispaniola [50–52]; these sequences were not included as background sequences in this analysis, since they did not meet the criteria for similarity to study sequences. In this study, sequences most similar to those from study participants in the DR were mostly from the United States, which may indicate that tourism plays a role in HIV transmission among FSWs in this region.

One limitation of this study is that it only included FSW with viral load >1,000 copies/mL; women with lower viral loads may also have had drug-resistant HIV. Also, this study used population sequencing to detect drug resistance mutations; these methods do not detect low-frequency drug resistance mutations, which can also contribute to treatment failure [53]. Another limitation is that INSTI resistance was only assessed among participants with MCR and not the whole cohort. In addition, the assay used for ARV drug testing only detects recent exposure to ARV drugs; ARV use outside of the detection window was not captured. For these reasons, the rates of ARV use and HIV drug resistance reported in this study should be considered to be minimum estimates. Finally, the qualitative drug assay does not provide information on ART adherence; results from quantitative ARV testing will be used to evaluate the relationship between ART adherence and HIV drug resistance in participants who had ARV drugs detected with viral loads >1,000 copies/mL.

## Conclusions

In summary, FSW are at heightened risk for HIV infection, are more likely to experience poor HIV outcomes, and play a critical role in the on-going global HIV epidemic. The findings of this report, including infrequent ART, high rates of resistance and MCR, and lack of viral suppression among many FSW using ARV drugs, highlight the urgent need for improved HIV care and treatment in this vulnerable population. Durable viral suppression would have health benefits for these women and would also reduce transmission to others, potentially reducing HIV incidence in the general population.

## Supporting information

S1 Table Factors associated with HIV drug resistance among HIV-positive female sex workers stratified by study site. The table shows results from univariate analyses of factors associated with resistance for each study site. Multivariate analyses were also performed by study site (not shown in the table). The initial model used for multivariate analysis included the following variables: country, age, being single, travel in the last 6 months, having ≥3 live births, visiting an ANC during last pregnancy, having >4 clients per week on average, consistent condom use, HIV duration, ART adherence level and detection of ARV drugs. Self-reported current ART correlated with self-reported adherence and therefore was not included in the initial multivariate model. Among 82 women from Tanzania, the following factors were independently associated with resistance: duration of HIV infection (odds ratio [OR], 95% confidence interval [CI]: 1.22, 1.01–1.47, p = 0.045), self-reported partial adherence (OR, 95% CI: 8.35, 1.66–42.03, p = 0.010), and detection of ARV drugs (OR, 95% CI: 18.41, 2.88–117.59, p = 0.002). Among 50 women from the DR, the following variables were independently associated with resistance: duration of HIV infection (OR, 95% CI: 1.15, 1.01–1.31, p = 0.032) and detection of ARV drugs (OR, 95% CI: 15.98, 2.80–91.33, p = 0.002). ^a^ The level of self-reported ART adherence was the sum of four adherence measures (adherence in last 4 days, always take on schedule, always follow instructions, and not skipped last weekend). Responses were scored as full adherence (4), partial adherence (1–3), no ART/no adherence (0). ^b^ This included nine participants who reported that they were “currently on ART”. ^c^ The stigma score was calculated as the sum of a 13-item Likert type scale [14]. Abbreviations: OR, odds ratio; CI, confidence interval; SD, standard deviation; ANC, antenatal clinic; STI, sexually transmitted infection; ART, antiretroviral treatment; ARV, antiretroviral.
(DOCX)
