# Supplementary material for: Antiretroviral drug use and HIV drug resistance in female sex workers in Tanzania and the Dominican Republic
Source: PLoS One. 2020 Oct 29;15(10):e0240890. doi: 10.1371/journal.pone.0240890 (PMC7595323; doi:10.1371/journal.pone.0240890)
Supplement: S1 Table — The table shows results from univariate analyses of factors associated with resistance for each study site. Multivariate analyses were also performed by study site (not shown in the table). The initial model used for multivariate analysis included the following variables: country, age, being single, travel in the last 6 months, having ≥3 live births, visiting an ANC during last pregnancy, having >4 clients per week on average, consistent condom use, HIV duration, ART adherence level and detection of ARV drugs. Self-reported current ART correlated with self-reported adherence and therefore was not included in the initial multivariate model. Among 82 women from Tanzania, the following factors were independently associated with resistance: duration of HIV infection (odds ratio [OR], 95% confidence interval [CI]: 1.22, 1.01–1.47, p = 0.045), self-reported partial adherence (OR, 95% CI: 8.35, 1.66–42.03, p = 0.010), and detection of ARV drugs (OR, 95% CI: 18.41, 2.88–117.59, p = 0.002). Among 50 women from the DR, the following variables were independently associated with resistance: duration of HIV infection (OR, 95% CI: 1.15, 1.01–1.31, p = 0.032) and detection of ARV drugs (OR, 95% CI: 15.98, 2.80–91.33, p = 0.002). a The level of self-reported ART adherence was the sum of four adherence measures (adherence in last 4 days, always take on schedule, always follow instructions, and not skipped last weekend). Responses were scored as full adherence (4), partial adherence (1–3), no ART/no adherence (0). b This included nine participants who reported that they were “currently on ART”. c The stigma score was calculated as the sum of a 13-item Likert type scale [14]. Abbreviations: OR, odds ratio; CI, confidence interval; SD, standard deviation; ANC, antenatal clinic; STI, sexually transmitted infection; ART, antiretroviral treatment; ARV, antiretroviral. (DOCX) [file pone.0240890.s001.docx]

**S1 Table. Factors associated with HIV drug resistance among HIV-positive female sex workers stratified by study site.**

| Characteristics | Tanzania | | | | Dominican Republic | | | |
| --- | --- | --- | --- | --- | --- | --- | --- | --- |
|  | **Total**  **n=88** | **Resistance detected** | | **P-value** | **Total**  **n=50** | **Resistance detected** | | **P-value** |
|  |  | **No**  **n=61** | **Yes**  **n=27** |  |  | **No**  **n=23** | **Yes**  **n=27** |  |
| Duration of infection, mean years (SD) | 3.0 (3.4) | 2.4 (3.0) | 4.9 (4.0) | **0.002** | 8.9 (6.0) | 6.9 (5.4) | 10.6 (6.0) | **0.029** |
| Currently on ART (self-report) |  |  |  | **<0.001** |  |  |  | **<0.001** |
| No | 49 (55.7%) | 45 (91.8%) | 4 (8.2%) |  | 13 (26.0%) | 13 (100.0%) | 0 (0.0%) |  |
| Yes | 39 (44.3%) | 16 (41.0%) | 23 (59.0%) |  | 37 (74.0%) | 10 (27.0%) | 27 (73.0%) |  |
| Self-reported adherence level^a^ |  |  |  | **<0.001** |  |  |  | **<0.001** |
| No ART/No adherence^b^ | 50 (56.8%) | 46 (92.0%) | 4 (8.0%) |  | 21 (42.0%) | 17 (81.0%) | 4 (19.0%) |  |
| Partial adherence | 26 (29.6%) | 10 (38.5%) | 16 (61.5%) |  | 13 (26.0%) | 2 (15.4%) | 11 (84.6%) |  |
| Full adherence | 11 (22.5%) | 5 (41.7%) | 7(58.3%) |  | 16 (32.0%) | 4 (25.0%) | 12 (75.0%) |  |
| ARV drugs detected in blood |  |  |  | **<0.001** |  |  |  | **<0.001** |
| No | 70 (79.6%) | 59 (84.3%) | 11 (15.7%) |  | 33 (66.0%) | 21 (63.6%) | 12 (36.4%) |  |
| Yes | 18 (20.5%) | 2 (11.1%) | 16 (88.9%) |  | 17 (34.0%) | 2 (11.8%) | 15 (88.2%) |  |
| Age in years, mean (SD) | 29.3 (7.0) | 27.4 (5.2) | 33.8 (8.48) | **<0.001** | 36.1 (9.5) | 33.9 (6.9) | 38.0 (11.0) | 0.13 |
| Marital status, single |  |  |  | **0.002** |  |  |  | 0.51 |
| No | 34 (38.6%) | 17 (50.0%) | 17 (50.0%) |  | 44 (88.0%) | 21 (47.7%) | 23 (52.3%) |  |
| Yes | 54 (61.4%) | 44 (81.5%) | 10 (18.5%) |  | 6 (12.0%) | 2 (33.3%) | 4 (66.7%) |  |
| Number of live births |  |  |  | **0.008** |  |  |  | 0.71 |
| 0 | 9 (10.2%) | 7 (77.8%) | 2 (22.2%) |  | 3 (6.0%) | 2 (66.7%) | 1 (33.3%) |  |
| 1-2 | 56 (63.6%) | 44 (78.6%) | 12 (21.4%) |  | 21 (42.0%) | 10 (47.6%) | 11 (52.4%) |  |
| 3+ | 23 (26.1%) | 10 (43.5%) | 13 (56.5%) |  | 26 (52.0%) | 11 (42.3%) | 15 (57.7%) |  |
| Seen provider at antenatal clinic last pregnancy |  |  |  | **0.045** |  |  |  | 0.27 |
| No | 22 (25.0%) | 19 (86.4%) | 3 (13.6%) |  | 1 (2.0%) | 1 (100.0%) | 0 (0.0%) |  |
| Yes | 66 (75.0%) | 42 (63.6%) | 24 (36.4%) |  | 49 (98.0%) | 22 (44.9%) | 27 (55.1%) |  |
| Travel (past 6 months) |  |  |  | 0.56 |  |  |  | 0.72 |
| No | 48 (54.4%) | 32 (66.7%) | 16 (33.3%) |  | 36 (72.0%) | 16 (44.4%) | 20 (55.6%) |  |
| Yes | 40 (45.5%) | 29 (72.5%) | 11 (27.5%) |  | 14 (28.0%) | 7 (50.0%) | 7 (50.0%) |  |
| Traveled for sex work (past 6 months) |  |  |  | 0.22 |  |  |  | 0.92 |
| No | 79 (90.8%) | 56 (70.9%) | 23 (29.1%) |  | 41 (82.0%) | 19 (46.3%) | 22 (53.7%) |  |
| Yes | 8 (9.2%) | 4 (50.0%) | 4 (50.0%) |  | 9 (18.0%) | 4 (44.4%) | 5 (55.6%) |  |
| Number of new/regular clients (past 30 days) |  |  |  | 0.31 |  |  |  | 0.11 |
| ≤4 | 55 (62.5%) | 36 (65.5%) | 19 (34.6%) |  | 14 (28.0%) | 9 (64.3%) | 5 (35.7%) |  |
| >4 | 33 (37.5%) | 25 (75.8%) | 8 (24.2%) |  | 36 (72.0%) | 14 (38.9%) | 22 (61.1%) |  |
| Inconsistent condom use with new/regular clients (past 30 days) |  |  |  | 0.63 |  |  |  | 0.11 |
| No | 39 (44.3%) | 26 (66.7%) | 13 (33.3%) |  | 45 (90.0%) | 19 (42.2%) | 26 (57.8%) |  |
| Yes | 49 (55.7%) | 35 (71.4%) | 14 (28.6%) |  | 5 (10.0%) | 4 (80.0%) | 1 (20.0%) |  |
| Alcohol (≥4 days per week) |  |  |  | 0.35 |  |  |  | 0.67 |
| No | 59 (67.1%) | 39 (66.1%) | 20 (33.9%) |  | 31 (62.0%) | 15 (48.4%) | 16 (51.6%) |  |
| Yes | 29 (33.0%) | 22 (75.9%) | 7 (24.1%) |  | 19 (38.0%) | 8 (42.1%) | 11 (57.9%) |  |
| Drug use ever |  |  |  | 0.88 |  |  |  | 0.62 |
| No | 82 (93.2%) | 57 (69.5%) | 25 (30.5%) |  | 28 (56.0%) | 12 (42.9%) | 16 (57.1%) |  |
| Yes | 6 (6.8%) | 4 (66.7%) | 2 (33.3%) |  | 22 (44.0%) | 11 (50.5%) | 11 (50.0%) |  |
| Sex work stigma^c^ |  |  |  | 0.72 |  |  |  | 0.49 |
| <36 | 61 (69.3%) | 43 (70.5%) | 18 (29.5%) |  | 30 (60.0%) | 15 (50.0%) | 15 (50.0%) |  |
| ≥36 | 27 (30.7%) | 18 (66.7%) | 9 (33.3%) |  | 20 (40.0%) | 8 (40.0%) | 12 (60.0%) |  |
| Gender-based violence (past 6 months) |  |  |  | 0.81 |  |  |  | 0.83 |
| No | 57 (64.8%) | 40 (70.2%) | 17 (29.8%) |  | 34 (68.0%) | 16 (47.1%) | 18 (52.9%) |  |
| Yes | 31 (35.2%) | 21 (67.7%) | 10 (32.3%) |  | 16 (32.0%) | 7 (43.8%) | 9 (56.3%) |  |

The table shows results from univariate analyses of factors associated with resistance for each study site. Multivariate analyses were also performed by study site (not shown in the table). The initial model used for multivariate analysis included the following variables: country, age, being single, travel in the last 6 months, having ≥3 live births, visiting an ANC during last pregnancy, having >4 clients per week on average, consistent condom use, HIV duration, ART adherence level and detection of ARV drugs. Self-reported current ART correlated with self-reported adherence and therefore was not included in the initial multivariate model. Among 82 women from Tanzania, the following factors were independently associated with resistance: duration of HIV infection (odds ratio [OR], 95% confidence interval [CI]: 1.22, 1.01-1.47, p=0.045), self-reported partial adherence (OR, 95% CI: 8.35, 1.66-42.03, p=0.010), and detection of ARV drugs (OR, 95% CI: 18.41, 2.88-117.59, p=0.002). Among 50 women from the DR, the following variables were independently associated with resistance: duration of HIV infection (OR, 95% CI: 1.15, 1.01-1.31, p=0.032) and detection of ARV drugs (OR, 95% CI: 15.98, 2.80-91.33, p=0.002).

^a^ The level of self-reported ART adherence was the sum of four adherence measures (adherence in last 4 days, always take on schedule, always follow instructions, and not skipped last weekend). Responses were scored as full adherence (4), partial adherence (1-3), no ART/no adherence (0).

^b^ This included nine participants who reported that they were “currently on ART”.

^c^ The stigma score was calculated as the sum of a 13-item Likert type scale [14].

Abbreviations: OR, odds ratio; CI, confidence interval; SD, standard deviation; ANC, antenatal clinic; STI, sexually transmitted infection; ART, antiretroviral treatment; ARV, antiretroviral.
